# Medicinal Properties of Matico

**Published:** 1847-04

**Authors:** 


*Medicinal properties of Matico.*—From a letter by Dr. Ruschenberger, U. S. N., to the Editor of the Boston Med. and Surg. Journal.

The *mactico, yuba del solado,* Piper Augustifolium, which has within two or three years past attracted the attention of the medical profession in England, was first brought to the United States by myself in 1834. It is said to have been accidentally discovered in 1824, in the battle of Ayacucho, by a soldier who was severely wounded, and in his anxiety to staunch the flow of blood, he pulled the leaves growing within his reach, and applied them to the wound, and the bleeding instantly ceased. He communicated the discovery to his wounded companions, who found its application equally efficacious. In Peru and Bolivia it became well known as a styptic, and has been externally used in the treatment of ulcers, but so far as I can learn, it has not been employed internally up to this time.

I have used it internally in tincture, two ounces to the pint, prepared by displacement ; in powder, in doses of a drachm mixed in a wine glass of water, repeated every two hours, in uterine hæmorrhage ; and in cold infusions (by displacement) half an ounce to the pint. It exerts a remarkably beneficial influence in menorrhagia, hæmatemesis, hæmoptysis, leucorrhœa, catarrhus vesicæ, and irritable bladder. It does not offend the stomach when given in powder or infusion (dose a wine-glassfull) ; in tincture, in drachm doses, is not complained of, but when carried to a half ounce I have seen it produce nausea. I have seen it arrest bleeding instantly from small arteries, even while the blood flowed in jets, and after lint, pressure, &c., had totally failed. To arrest the bleeding from leech bites, and the troublesome hæmorrhage which sometimes follows the extraction of a tooth, I think it will be found a very certain remedy. When used to arrest bleeding, it should be in coarse powder and moistened with cold water. A strong tincture, four ounces to the pint, might possibly answer. The taste is not unpleasant.

This is a hasty outline of my experience, which is confirmed by that of Dr. Munro, of Dundee ; Dr. Jeffreys, of Liverpool ; and Dr. Hunter Lane, of lancaster, as you may see by reference to the 8th part of Braithwaite's Retrospect of Practical Medidine and Surgery (1844, New York) page 37. You will find a notice of the article in the second American edition of Pereira's Materia Medica, edited by Professor Carson, vol. 2, page 222.

There is, is believe, no matico in the United States on sale at this time, although the Medical Journals contain occasional notices of its employment in England. I am fully persuaded that its virtues are such as to warrant me in reccommending it to the profession for examination and trial. A part of what I have recently received I have forwarded to you.

There are two varieties of matico known ; one is the matico hoja redonda (round leaved matico) ; and the other, matico hoja puntiaguda (pointed leaved.) The latter is considered the best, and is the kind sent to you.

Specimens of matico have been presented by me to several medical friends in Philadelphia.

